# Factors associated with falls among older adults living in institutions

**DOI:** 10.1186/1471-2318-13-6

**Published:** 2013-01-15

**Authors:** Javier Damián, Roberto Pastor-Barriuso, Emiliana Valderrama-Gama, Jesús de Pedro-Cuesta

**Affiliations:** 1 National Center for Epidemiology, Carlos III Institute of Health, Madrid, Spain; 2 Consortium for Biomedical Research in Neurodegenerative Diseases (Centro de Investigación Biomédica en Red sobre Enfermedades Neurodegenerativas - CIBERNED), Madrid, Spain; 3 Consortium for Biomedical Research in Epidemiology and Public Health (CIBER en Epidemiología y Salud Pública - CIBERESP), Madrid, Spain; 4 "Arroyo de la Media Legua" Primary Care Center, Madrid Health Service (Servicio Madrileño de Salud), Madrid, Spain; 5 Centro Nacional de Epidemiología. Instituto de Salud Carlos III, Av/ Monforte de Lemos 5, 28029, Madrid, Spain

**Keywords:** Accidental falls, Epidemiology, Residential facilities, Nursing homes, Comorbidity

## Abstract

**Background:**

Falls have enormous impact in older adults. Yet, there is insufficient evidence regarding the effectiveness of preventive interventions in this setting. The objectives were to measure the frequency of falls and associated factors among older people living institutions.

**Methods:**

Data were obtained from a survey on a probabilistic sample of residents aged ≥65 years, drawn in 1998-99 from institutions of Madrid (Spain). Residents, their caregivers, and facility physicians were interviewed. Fall rates were computed based on the number of physician-reported falls in the preceding 30 days. Adjusted rate ratios were computed using negative binomial regression models, including age, sex, cognitive status, functional dependence, number of diseases, and polypharmacy.

**Results:**

The final sample comprised 733 residents. The fall rate was 2.4 falls per person-year (95% confidence interval [CI], 2.04-2.82). The strongest risk factor was number of diseases, with an adjusted rate ratio (RR) of 1.32 (95% CI, 1.17-1.50) for each additional diagnosis. Other variables associated with falls were: urinary incontinence (RR = 2.56 [95% CI, 1.32-4.94]); antidepressant use (RR = 2.32 [95% CI, 1.22-4.40]); arrhythmias (RR = 2.00 [95% CI, 1.05-3.81]); and polypharmacy (RR = 1.07 [95% CI, 0.95-1.21], for each additional medication). The attributable fraction for number of diseases (with reference to those with ≤ 1 condition) was 84% (95% CI, 45-95%).

**Conclusions:**

Number of diseases was the main risk factor for falls in this population of institutionalized older adults. Other variables associated with falls, probably more amenable to preventive action, were urinary incontinence, antidepressants, arrhythmias, and polypharmacy.

**Virtual slides:**

The virtual slide(s) for this article can be found here:

http://www.diagnosticpathology.diagnomx.eu/vs/3916151157277337

## Background

Unintentional injuries are estimated to be the fifth leading cause of death in older adults, with falls accounting for two-thirds of such deaths [1]. In nursing homes, fall rates are more than twice as high compared to non-institutionalized populations [2], are associated with poorer survival [3] and constitute a considerable financial burden. [4,5] Falls are the main cause of the recently reported increasing trend in traumatic spinal cord injury incidence among older adults [6]. Furthermore, non-injurious falls adversely affect quality of life, induce fear of falling (with its own serious consequences [7]), and limit mobility and activity [2,8]. The prevalence of risk factors is higher in nursing homes and most residents have more than one risk factor [9]. In the long-term care setting well established risk factors are muscular weakness, balance and gait deficits, poor vision, delirium, cognitive and functional impairment, orthostatic hypotension, urinary incontinence, medications (number of drugs, antidepressants, psychotropics, non-steroidal anti-inflammatory drugs, vasodilators) [9,10] and comorbidities (depression, stroke, Parkinson disease, arthritis) [9,10]. A recent large study in 528 German nursing homes found than about 75% of falls occurred in the resident’s rooms or in the bathrooms, with transfers and walking responsible for 41% and 36% of all falls respectively [11]. Systematic reviews have found inconsistent results regarding effectiveness of interventions to prevent falls in care homes. A review found insufficient evidence [12], but another recent systematic review found that some interventions (prescription of vitamin D) and some multifactorial interventions, can be effective [13]. The targets are multiple and varied, thus we believe that non-experimental studies, conducted on representative populations, can provide useful clues in the study of this problem and better inform prevention strategies. Accordingly, our study sought to measure fall rates and potential associations between falls and a comprehensive list of -essentially predisposing- factors, among institutionalized older persons. We also present estimates stratified by severity of falls.

## Methods

### Sampling

Data were obtained from a survey conducted in the period June 1998 through June 1999 on a probabilistic sample of residents aged 65 years and older, drawn from public and private facilities situated in or within a 35-kilometre radius of the city of Madrid (Spain). Study participants were selected through stratified cluster sampling, with one stratum including 22 public and 25 subsidized (i.e., privately owned but publicly funded) facilities, and the other stratum including 139 private institutions. First, we sampled 25 public/subsidized and 30 private institutions, with probability proportional to size. Then we randomly sampled 10 men and 10 women from each public/subsidized facility selected, and 5 men and 5 women from each private facility selected. Four private facilities declined to participate (totaling potential 40 subjects), and 45 additional residents could not be selected due to absence or refusal, yielding an overall response rate of 89% (715/800). Thirty-nine subjects were replaced by a resident of the same facility and sex, giving a total of 754 structured interviews. Ten subjects with a stay of less than 30 days were excluded, mainly because falls refer to the previous 30 days.

### Data-collection and variable definition

Using structured questionnaires, purpose-trained geriatricians or residents in geriatrics collected data by interviewing the residents, their main caregivers, and the facility's physician (or nurse).

*Falls*: physicians (or nurses in 8% of cases) were asked about the number of falls experienced by a given resident in the preceding month. Interviews were conducted with the aid of the medical records and nursing annotations. Where the answer was one or more, they were then asked to state whether any falls had had any of the following consequences: open wound; hip fracture; other fracture; cerebral hemorrhage; or transfer to hospital. Fallers with any of these reported consequences were classified into severe (or non-severe otherwise).

*Socio*-*demographic variables*: residents' sex, age, marital status, and educational level were obtained.

*Medical conditions and medications*: physicians (or nurses in 8% of cases) were asked whether any resident had suffered from a list of diseases (see below), and the number of diseases was then computed (minus 1 for subjects suffering from a specific disease in analyses involving that particular disease). Interviewers recorded all medications used in the preceding 7 days. Thereafter we computed the total number of medications and registered the use of antidepressants (World Health Organization ATC code: N06A), anxiolytics (N05B), hypnotics (N05C), and antipsychotics (N05A).

*Functional status*: we used the Barthel Index, as modified by Shah et al. [14] Subjects (55%) or their main caregivers (45%) were asked as to the residents’ degree of dependence in performing the following basic activities of daily living (ADL): eating; going to the toilet; personal hygiene; bathing/showering; dressing/undressing; transferring; walking; use of stairs; and urinary/faecal continence. For each activity there are five response levels, which can be scored with diverse points ranging 0–5, 0–10 or 0–15 depending on the activity, resulting in a overall range of 0 (total dependence) to 100 (totally independent) points, with 1-point increments.

*Cognitive status*: we used a Spanish version of Pfeiffer's Short Portable Mental Status Questionnaire [15] (range 0–10 errors, allowing for one more or one less error if subject has had only a grade school education or has had beyond high school education, respectively), suitably amended to adapt to the institutional setting. For logistic reasons, Pfeiffer's test could only be administered to 460 residents. Assuming this latter group to be basically a random sample (missing at random) and deeming this variable of sufficient relevance for most analyses, we decided to use multiple imputation [16,17]. We obtained 5 Pfeiffer-score imputed data sets using a cumulative-odds, ordinal logistic model that included age, sex, education, number of falls, Barthel Index (as restricted quadratic splines), number of diseases, number of medications, dementia status, and variables relating to the survey design (cluster and strata identifiers, and sampling weights).

*Urinary incontinence*: residents or their caregivers were asked about the occurrence of any leakage in the preceding 2 weeks.

*Vision and hearing*: these conditions were assessed by means of the two pertinent, four-category, minimum-data-set questions [18], and then dichotomized as good/mild versus moderate/severe impairment. Caregivers or residents were asked to assess: residents' ability to see in adequate light, with glasses where used; and hearing status, even with a hearing aid where used.

*Use of restraints and behavioral problems*: residents' main caregivers were asked about the use of physical restraints and the occurrence of behavioral problems (wandering, physically abusive or socially inappropriate behavior) in the preceding 7 days.

*Assistive devices*: subjects or their caregivers were asked about the use of a cane or walker in the previous week. Similarly, subjects or their caregivers were also asked about the occurrence of *insomnia* in the past seven days.

*Self*- *and physician*-*rated health*: subjects were asked about their health via the question, "In general terms, how would you describe your health: very good; good; fair; poor; or very poor?" For study purposes, we used a dichotomized version (very good/good, and fair/poor/very poor). *Physicians* were asked to rate residents' health in a similar fashion.

*Depressive symptoms*: using a 10-item version of the Geriatric Depression Scale [19], scored from 0-10, residents were asked to respond with reference to their status during the previous 7 days.

### Ethics statement

Informed consent was obtained verbally from the study subject or their next of kin. The written consent was not requested taking into account the observational nature of the whole study and trying to minimize any influence in the results. The informed consent, however, was documented in the Case Report sheets. These, containing the subjects’ names, were kept apart, leaving questionnaires and datasets without names. The “Carlos III” Institute of Health Research Committee approved the study, which met the legal requirements in Spain since, in the case of non-experimental research there was no legal requirement for an ethics committee report at that time.

### Analysis

Fall rates were computed as number of falls over number of study subjects (each study subject contributing 30 person-days), and then converted into rates per person-year. Risks were computed with the exponential formula [20]: Risk = 1 − exp(−rate × time). For each determinant we further computed the age-, sex-, cognition- and functional dependence-adjusted rate ratios (RR) and their 95% confidence intervals (CI) for falls in the preceding 30 days, using negative binomial regression models [21]. Cognition (Pfeiffer’s score) and functional dependence (Barthel Index score) were included in the models as restricted quadratic splines [22]. Associations for urinary incontinence included a customized Barthel Index with the continence items eliminated. A second set of models was additionally adjusted for number of diseases, and number of medications. To investigate the effect of variables on severity, we fitted polytomous logistic regression models with 3 outcome categories, namely: non-fall (base outcome); non-severe fall; and severe fall. Moreover, these latter models may help explore potential reverse causation, e.g. the determinant value can be a consequence of the fall. This phenomenon is less plausible if the estimate associated with serious falls fails to exceed that for non-severe falls, since there are fewer instances in which non-severe falls can modify the specific risk-factor status. All analyses involving Pfeiffer scores comprised pooled estimates of the 5 imputed datasets, with standard errors computed by taking the within- and between-imputation components into account [16]. To provide an approximation of the population impact of the most relevant factors, we computed attributable fractions for recurrent events [23], using the formula *AFr* = *P*(*RR*_*a*_ - 1)/*RR*_*a*_, where *P* is the proportion of exposed events (number of falls among exposed subjects/total number of falls), and *RR*_*a*_ is the fully adjusted rate ratio. For confidence intervals we used the formula proposed by Greenland [24]. *AFr* quantifies the proportion of total falls in the population attributable to the exposure. All analyses were weighted to the underlying population distribution to re-establish proportionality in sex and type of facility, and accounted for the effect of stratification and clustering on point and interval estimates. Absolute numbers represent empirical (i.e., not weighted) observations. Analyses were performed using the Stata 11 software program [25].

## Results

Of the 744 residents with stays longer than 30 days, data were obtained on falls among 733. Table 1 shows the basic characteristics of the population. Residents had a mean age of 83.4 years (95% CI, 82.6-84.1), a mean of 3.2 diseases (95% CI, 2.9-3.5), and a mean of 4.2 medications (95% CI, 3.9-4.5). Detailed information on the grouping of study subjects and the proportion of fallers by subgroup are showed in an additional table [see Additional file 1: Table S1]. The weighted fraction of residents reporting at least 1 fall in the preceding 30 days was 12% (95% CI, 9-15%). One quarter of those who fell suffered adverse outcomes, comprising the severe-fall group. Among fallers, 68%, 21%, and 12% had 1, 2, and ≥3 falls respectively. The total number of falls was 146, corresponding to a rate of 2.4 (95% CI, 2.04-2.82) falls per person-year. The rate of at least one fall was 1.5 (95% CI, 1.22-1.84) per person-year, which translated as a 1-year risk of falling of 1 − exp(−1.5) = 0.78.

**Table 1:** Basic characteristics of study participants

**Variables**	**No**. (%) ^**a**^
Sex	
Women	408 (76)
Men	325 (24)
Age (years)	
65-74	92 (12)
75-84	301 (41)
≥ 85	326 (47)
Facility ownership	
Public	404 (45)
Subsidized	78 (9)
Private	251 (47)
Functional dependence	
Independent (100^b^)	186 (22)
Mild (91-99)	177 (25)
Moderate (61-90)	152 (23)
Severe (21-60)	91 (15)
Total (0-20)	109 (16)
Cognitive status^c, d^	
Normal (≤2)	269 (54)
Mild (3-4)	65 (14)
Moderate (5-7)	60 (15)
Severe (≥8)	60 (17)

^a^ Unweighted counts and weighted percentages.

^b^ Barthel Index score.

^c^ Empirical number of observations and weighted percentages computed through multiple imputation.

^d^ Pfeiffer's Short Portable Mental Status Questionnaire score.

**Figure 1:**
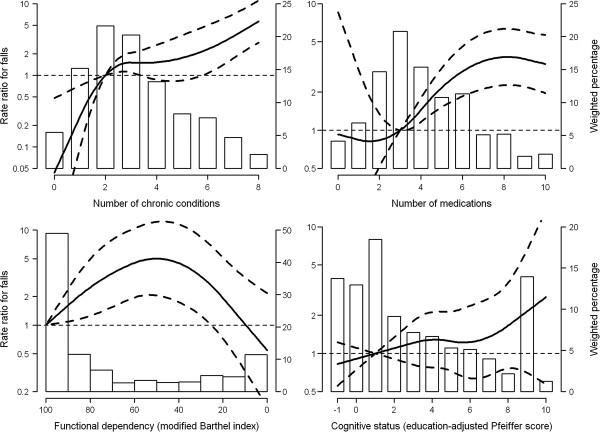
**Rate ratios for falls by number of chronic conditions**, **number of medications**, **functional dependency**, **and cognitive status in institutionalized older people of Madrid**, **Spain**, **1998**–**1999**. Curves represent adjusted rate ratios (solid lines) and their 95% confidence intervals (dashed lines) based on restricted quadratic splines with knots at 1, 3, and 7 chronic conditions; 1, 4, and 9 medications; 90, 60, and 20 points for the modified Barthel index; and 3, 5, and 8 education-adjusted errors for the Pfeiffer’s questionnaire. The reference value (rate ratio = 1) was set at 2 chronic conditions, 3 medications, 100 points for the modified Barthel index, and 1 education-adjusted error for the Pfeiffer’s questionnaire. Rate ratios were adjusted for sex, age, functional dependency, and cognitive status. Bars represent the weighted bar charts of the number of chronic conditions, the number of medications, and the education-adjusted Pfeiffer score, as well as the weighted histogram of the modified Barthel index.

Table 2 shows rate ratio estimates for selected variables. Strong associations were found for number of diseases (RR = 1.40, for an increase of 1 disease). Figure 1 shows better this association. The increase in risk is very strong in the first section, up to 2-3 diseases. From that point the slope is much less pronounced. A strong association was also found for polypharmacy (RR = 1.19, for an increase of 1 drug; Table 2). In this case, the Figure 1 shows a flat association in the first section followed by a sudden elevation in risk starting at 3 drugs and rising until 8 drugs. When these variables were mutually adjusted, number of diseases remained strong (RR = 1.32) and the effect of polypharmacy weakened (RR = 1.07). Regarding a possible interaction between both, we included a product term consisting of number of medications multiplied by dichotomized number of diseases. The adjusted rate ratios (95% CI) for an increase of 1 medication were 1.31 (1.05-1.65) and 1.14 (1.02-1.27) for those with 0-1 and ≥2 conditions respectively (*P* value for homogeneity = 0.25). Among psychotropic medications, antidepressants displayed a marked increased risk (RR = 3.40) with an equally plausible effect for anxiolytics (RR = 1.64). All these effects were diluted when additionally adjusted for number of diseases and polypharmacy. Functional dependence was also associated with falls, with rate ratios increasing with dependence and decreasing in the last category of total dependence. The Figure allows a better appraisal of this variable's behavior. Fall rate ratios increased with level of cognitive impairment but the association was imprecise, i.e. wide confidence intervals (Table 2 and Figure 1). Urinary incontinence displayed a clear effect in both models (RRs: 2.89 and 2.56).

**Table 2:** **Association between selected variables and falls among institutionalized older adults in Madrid**, **Spain**

**Variables**	**Crude RR**	**RR**^**a**^ (**95**% **CI**)	**RR**^**b**^ (**95**% **CI**)
Male gender	1.21	1.37 (0.74-2.53)	1.12 (0.60-2.07)
Age (per 5 y)	1.02	0.95 (0.81-1.11)	0.86 (0.71-1.03)
Marital status (no spouse)	1.00	1.27 (0.72-2.26)	1.71 (0.93-3.17)
Education			
Less than primary	1.00	1.00	1.00
Primary	1.27	1.22 (0.63-2.37)	1.06 (0.53-2.12)
Secondary or higher	1.30	1.09 (0.49-2.45)	1.17 (0.54-2.52)
Length of stay (years)			
0-2	1.00	1.00	1.00
3-5	1.05	1.11 (0.61-2.02)	1.34 (0.72-2.50)
≥ 6	1.38	1.50 (0.78-2.87)	1.31 (0.68-2.53)
Facility size (per 50 beds)	1.01	1.03 (0.94-1.13)	1.01 (0.93-1.10)
Facility			
Public	1.00	1.00	1.00
Subsidized	1.69	1.14 (0.59-2.20)	1.41(0.62-3.20)
Private	1.29	1.02 (0.49-2.14)	1.13 (0.57-2.23)
No. of diseases (per 1)	1.40	1.40 (1.27-1.54)	1.32 (1.17-1.50)
No. of medications (per 1)	1.27	1.19 (1.06-1.33)	1.07 (0.95-1.21)
Antidepressant use	3.30	3.40 (1.65-7.04)	2.32 (1.22-4.40)
Anxiolytic use	1.73	1.64 (0.80-3.36)	1.48 (0.75-2.91)
Hypnotic use	0.56	0.50 (0.23-1.08)	0.62 (0.24-1.56)
Antipsychotic use	1.26	0.73 (0.36-1.49)	0.92 (0.47-1.82)
Functional dependence			
Independent (100^c^)	1.00	1.00	1.00
Mild (91-99)	2.24	2.16 (0.70-6.60)	1.89 (0.60-5.97)
Moderate (61-90)	3.58	2.76 (1.19-6.37)	2.39 (0.87-6.55)
Severe (21-60)	6.17	5.03 (1.77-14.28)	3.02 (1.17-7.78)
Total (0-20)	1.83	0.89 (0.24-3.25)	0.86 (0.26-2.82)
Use of cane	1.59	2.07 (0.93-4.61)	1.55 (0.79-3.07)
Use of walker	2.29	1.33 (0.63-2.80)	0.91 (0.46-1.82)
Behavioural problems	1.98	1.32 (0.64-2.74)	1.09 (0.56-2.11)
Cognitive status			
Normal (≤2^d^)	1.00	1.00	1.00
Mild (3–4)	1.10	0.77 (0.35-1.70)	0.78 (0.33-1.83)
Moderate (5–7)	1.52	0.90 (0.42-1.94)	0.68 (0.29-1.60)
Severe (≥8)	1.77	1.85 (0.58-5.87)	2.15 (0.82-5.65)
Vision (moderate/severe)	1.22	0.89 (0.51-1.57)	0.90 (0.55-1.47)
Hearing (moderate/severe)	0.97	0.90 (0.38-2.13)	1.11 (0.49-2.54)
Insomnia	1.21	1.16 (0.63-2.15)	0.88 (0.50-1.55)
Urinary incontinence	3.42	2.89 (1.48-5.65)	2.56 (1.32-4.94)
Physical restraint	1.11	0.74 (0.28-1.93)	0.63 (0.29-1.36)
Self-rated health (fair/poor/very poor)	2.00	1.65 (0.93-2.95)	1.34 (0.74-2.41)
Physician-rated health (fair/poor/very poor)	2.31	2.11 (1.18-3.78)	0.97 (0.55-1.71)
Depressive symptoms (per 1 GDS point)	1.14	1.06 (0.95-1.19)	1.01 (0.92-1.11)

*Abbreviations*: *CI* = confidence interval; *GDS* = Geriatric Depression Scale; *RR* = rate ratio.

^a^ Rate ratio adjusted for age, sex, cognitive status, and functional dependence.

^b^ Additionally adjusted for number of diseases and number of medications (for psychotropic the particular index drug was excluded from the count).

^c^ Barthel Index score.

^d^ Pfeiffer's Short Portable Mental Status Questionnaire score.

Results for selected medical conditions are shown in Table 3. Clear associations were observed for arrhythmias, anemia, peripheral arterial disease, cancer, obstructive pulmonary disease, anxiety, and arthritis. When additional adjustment was made for number of diseases and polypharmacy, many associations became doubtful although some are worth considering (arrhythmias, anxiety, depression, peripheral arterial disease, and obstructive pulmonary disease). In the case of depression and anxiety, however, almost the entire effect was explained by antidepressant and anxiolytic use. The estimates differentiated by fall severity show no clear superior effect for severe falls in any variable (Table 4) but, due to the limited statistical power of homogeneity tests, some differences are worth mentioning. Antidepressants, use of cane and insomnia may have a stronger effect with non-severe falls, whereas obstructive pulmonary disease and hypertension may have a stronger effect with severe falls. In addition to the last two diseases probable risk factors for severe falls may encompass: Number of diseases, polypharmacy, cancer, arrhythmias, peripheral arterial disease, and arthritis.

**Table 3:** **Association between prevalent diseases and falls among institutionalized older adults in Madrid**, **Spain**

**Disease** (**prevalence**, %)	**Crude RR**	**RR**^**a**^ (**95**% **CI**)	**RR**^**b**^ (**95**% **CI**)
Cancer (8.7)	2.47	2.89 (1.21-6.91)	1.58 (0.70-3.55)
Obstructive pulmonary disease (19.3)	2.37	2.78 (1.47-5.23)	1.60 (0.91-2.81)
Arrhythmias (22.3)	3.10	3.36 (1.80-6.30)	2.00 (1.05-3.81)
Hypertension (45.1)	1.53	1.54 (0.86-2.75)	1.03 (0.59-1.82)
Ischemic heart disease (16.8)	1.12	0.94 (0.50-1.76)	0.62 (0.33-1.18)
Congestive heart failure (20.2)	2.43	2.16 (1.15-4.04)	0.99 (0.59-1.67)
Peripheral arterial disease (26.9)	2.52	2.91 (1.62-5.20)	1.62 (0.93-2.82)
Stroke in past year (6.4)	1.66	1.96 (0.50-7.80)	1.79 (0.28-11.59)
Diabetes (17.6)	1.31	1.09 (0.58-2.03)	0.87 (0.53-1.43)
Anemia (17.8)	2.86	2.84 (1.49-5.41)	1.54 (0.85-2.79)
Alzheimer's disease (14.1)^c^	1.25	0.92 (0.42-2.06)	1.56 (0.67-3.66)
Other dementias (20.6)^c^	1.30	1.09 (0.56-2.12)	1.21 (0.65-2.25)
Parkinson's disease (7.7)	1.91	1.14 (0.57-2.29)	1.09 (0.57-2.09)
Epilepsy (3.9)	1.41	1.24 (0.48-3.18)	0.80 (0.35-1.84)
Depression (21.0)	2.31	2.49 (1.38-4.50)	1.55 (0.95-2.51)
Anxiety disorder (26.7)	2.34	2.39 (1.38-4.15)	1.75 (0.88-3.46)
Arthritis (34.3)	1.83	1.80 (0.95-3.41)	1.65 (0.85-3.22)

*Abbreviations*: *CI* = confidence interval; *RR* = rate ratio.

^a^ Rate ratio adjusted for age, sex, cognitive status, and functional dependence.

^b^ Additionally adjusted for modified number of diseases, and number of medications.

^c^ These models did not include cognitive status.

**Table 4:** **Association of variables with risk of any fall**, **by severity of fall**, **among institutionalized older adults in Madrid**, **Spain**

	**Non**-**severe**	**Severe**	
Variables	OR^a^ (95% CI)	OR^a^ (95% CI)	*P* value ^b^
Male gender	0.99 (0.54-1.81)	1.25 (0.54-2.88)	0.60
Age (per 5 y)	0.92 (0.72-1.17)	1.00 (0.72-1.38)	0.67
No spouse	1.25 (0.60-2.62)	2.60 (0.62-10.87)	0.28
Education			0.54
Primary	1.61 (0.81-3.20)	0.85 (0.31-2.32)	
Secondary or higher	1.47 (0.54-3.99)	1.08 (0.29-4.03)	
Length of stay (years)			0.95
0-2	1.00	1.00	
3-5	1.27 (0.58-2.79)	1.01 (0.30-3.42)	
≥ 6	1.41 (0.61-3.24)	1.22 (0.37-4.05)	
Facility size (per 50 beds)	1.08 0.99-1.17)	1.12 (0.95-1.32)	0.58
Facility ownership			0.52
Public	1.00	1.00	
Subsidized	0.74 (0.35-1.55)	1.07 (0.31-3.69)	
Private	0.64 (0.31-1.33)	0.43 (0.14-1.38)	
No. of diseases	1.40 (1.26-1.55)	1.47 (1.17-1.84)	0.68
No. of medications	1.31 (1.19-1.44)	1.17 (1.01-1.36)	0.22
Antidepressant use	3.94 (1.87-8.30)	1.28 (0.29-5.64)	0.16
Anxiolytic use	1.53 (0.74-3.20)	0.84 (0.27-2.63)	0.22
Hypnotic use	0.60 (0.19-1.88)	0.41 (0.07-2.28)	0.69
Antipsychotic use	0.99 (0.37-2.68)	0.71 (0.17-3.01)	0.69
Functional dependence			0.89
Independent (100^c^)	1.00	1.00	
Mild (91-99)	1.15 (0.44-2.97)	2.50 (0.21-29.45)	
Moderate (61-90)	2.44 (0.80-7.42)	4.72 (0.78-28.49)	
Severe/Total (0-60)	2.79 (0.78-10.01)	6.67 (0.89-49.95)	
Use of cane	2.21 (1.02-4.82)	0.85 (0.28-2.62)	0.14
Use of walker	1.22 (0.49-3.06)	2.16 (0.61-7.68)	0.47
Behavioral problems	1.30 (0.53-3.19)	0.76 (0.23-2.47)	0.46
Cognitive status			0.55
Normal (≤2^d^)	1.00	1.00	
Mild (3-4)	0.77 (0.26-2.28)	1.64 (0.39-6.99)	
Moderate (5-7)	0.91 (0.37-2.19)	0.34 (0.05-2.39)	
Severe (≥8)	1.84 (0.47-7.23)	1.46 (0.31-7.01)	
Vision problems	1.63 (0.82-3.26)	0.68 (0.11-4.15)	0.37
Hearing problems	0.95 (0.29-3.14)	1.80 (0.45-7.10)	0.43
Insomnia	1.91 (0.92-3.96)	0.78 (0.28-2.15)	0.18
Urinary incontinence	2.74 (1.42-5.30)	1.34 (0.49-3.66)	0.20
Physical restraint	0.88 (0.30-2.56)	1.00 (0.27-3.75)	0.85
Poor self-rated health	1.76 (0.95-3.28)	1.32 (0.49-3.55)	0.65
Poor physician-rated health	2.20 (1.24-3.90)	2.77 (0.78-9.81)	0.73
Depressive symptoms	1.17 (1.03-1.33)	1.14 (0.94-1.38)	0.82
Cancer	2.46 (0.99-6.14)	2.94 (0.97-8.92)	0.77
Obstructive pulmonary disease	1.77 (0.88-3.57)	4.26 (1.35-13.46)	0.19
Arrhythmias	2.48 (1.13-5.47)	3.46 (1.32-9.02)	0.54
Hypertension	1.18 (0.61-2.30)	3.02 (1.05-8.68)	0.13
Ischemic heart disease	1.19 (0.51-2.79)	0.79 (0.23-2.74)	0.59
Congestive heart failure	1.73 (0.89-3.35)	1.75 (0.63-4.87)	0.98
Peripheral arterial disease	1.93 (1.06-3.49)	3.23 (1.23-8.52)	0.34
Stroke in past year	2.18 (0.64-7.46)	0.42 (0.05-3.83)	0.22
Diabetes	1.24 (0.58-2.64)	1.09 (0.33-3.62)	0.87
Anemia	2.55 (1.26-5.17)	1.67 (0.75-3.74)	0.40
Alzheimer's disease	0.94 (0.39-2.29)	0.23 (0.02-2.36)	0.20
Other dementias	0.94 (0.39-2.23)	1.41 (0.51-3.89)	0.43
Parkinson's disease	1.63 (0.69-3.84)	0.84 (0.18-3.95)	0.47
Epilepsy	1.64 (0.52-5.16)	3.89 (0.82-18.47)	0.34
Depression	3.70 (1.95-7.03)	1.80 (0.64-5.02)	0.20
Anxiety disorder	2.51 (1.50-4.20)	2.04 (0.90-4.64)	0.65
Arthritis	1.65 (0.85-3.19)	3.68 (1.00-13.58)	0.24

*Abbreviations*: *CI* = confidence interval; *OR* = odds ratio.

^a^ Odds ratio of suffering at least one fall, adjusted for age, sex, cognitive status, and functional dependence. The reference base outcome group is non-fallers.

^b^ Two-sided tests of the equality of non-severe and severe fall coefficients.

^c^ Barthel Index score. Severe and total categories were collapsed due to small numbers

^d^ Pfeiffer's Short Portable Mental Status Questionnaire score.

In terms of potential population impact, higher attributable fractions for relevant variables were as follows: number of diseases (dichotomized, with reference to those with ≤ 1 condition), 84% (95% CI, 45-95%); urinary incontinence, 49% (95% CI, 20-67%); arrhythmias, 24% (95% CI, 4-40%); and antidepressants, 17% (95% CI, 5-27%).

## Discussion

The main finding in this study on institutionalized older adults was the very strong association between multiple diseases and the fall rate. We also found clear associations with other factors consistently reported in the literature, such as urinary incontinence and antidepressant use. Among specific medical conditions, arrhythmias showed the clearest association. Although we were unable to address environmental or staff determinants, numerous personal characteristics were studied in sufficient depth, mostly linked to health status (patient-related or intrinsic factors). Our fall rate was similar to that reported elsewhere in comparable settings [11,26-28].

### Number of diseases

Number of diseases remained a very strong risk factor in all models. Although we feel that this effect may well reflect the sum of causal contributors inherent in the different diseases, considering some type of cumulative component leading to weakness and frailty is nonetheless compelling (and in line with a proposed approach to the concept and measurement of frailty [29]). However, it should be noted that the great part of the increase in risk occurs when comparing people from 0-1 conditions (whose risk is certainly very low) to 2-3 diseases. From there on, the increase in risk is less marked. The role of polypharmacy further complicates these issues, as it may act as both a confounder and an intermediate factor. As regards the interaction between these two variables, we found that polypharmacy was a stronger risk factor for falls in those with 0 or 1 chronic condition versus those with a higher number of diseases (we opted to measure the effect of polypharmacy, as this is the variable with more practical interest in terms of potential for prevention in this setting). Even so, the absolute benefit of reducing polypharmacy is expected to be notably greater in the group with higher number of diseases because the fall rates are clearly higher in this group. For comparison purposes, we made a search for other studies conducted in an institutional setting with data on number of diseases but were unable to locate more than one, which reported a null association with number of diseases [30]. Among community-dwelling older people, however, a study on women reported results very similar to ours, including the role of polypharmacy and its interaction with number of conditions [31].

### Psychoactive drugs

A clear association was solely observed with antidepressants. One systematic review on psychoactive drugs and fall risk in nursing homes reported strong evidence on the association with multiple drugs, antidepressants, and anti-anxiety medications [32]. In addition, various studies found consistently clear relations between antidepressants – whether tricyclic or selective serotonin-reuptake inhibitors (SSRI) – and risk of falls [33]. While the mechanism is not clear in the case of the latter type, it has been hypothesized that SSRI may, like tricyclics, also have cardiovascular effects [34]. It has also been suggested that SSRI might induce urinary incontinence [35], which is a solid risk factor for falls. Antidepressants, albeit useful, are consistently associated with risk of falling, something that should therefore be borne in mind and the necessity of their use be periodically questioned in each particular situation. These can be conflicting issues in view of the determinant role of depression in older persons' state of health and quality of life. Recent research has found that antidepressants are effective (beyond placebo) solely in cases of severe depression, thus calling into question their effectiveness in milder cases [36]. This ought to be taken into account in the management of depressive symptoms.

### Functioning and cognition

Residents with intermediate levels of functioning registered the highest risk, with similar [37] or equivalent [38,39] results being reported by other studies. Another study found results compatible with the same idea (with fall rates lowest in those with the best and worst balance) and also a higher incidence of injurious falls for those who could stand unaided [26]. This pattern is only to be expected, since persons with severe functional limitations are usually less exposed to such risks. With respect to cognition, the risk function displayed a pattern of continuous increase in rate ratios as the level of impairment increased, but with substantial statistical imprecision.

### Severe falls

It has been stated that even more important than identifying risk factors for falling is identifying risk factors for injurious falls, because most falls do not result in injury [2]. In our population one quarter of those who fell had important consequences (open wound; hip fracture; other fracture; cerebral hemorrhage; or transfer to hospital). Thus, considering the very high fall rates, we believe falling is a serious problem. We found potential risk factors for severe falls, namely: number of diseases, polypharmacy, cancer, obstructive pulmonary disease, arrhythmias, hypertension, peripheral arterial disease, and arthritis. On the other hand – and with the caution that wide confidence intervals recommend – it would appear that patients with Alzheimer’s disease (OR = 0.23) and perhaps also stroke patients (OR = 0.42) could be protected from severe falls. Rubenstein and Josephson describe a review of risk factors for injurious falls among nursing home residents: Lower extremity weakness, female gender, poor vision and hearing, disorientation, number of falls, impaired balance, dizziness, low body mass and use of mechanical restraints [2]. Our results are not consistent with such health conditions. Nevertheless, the above mentioned disorders (including its associated medications, e.g. antihypertensives or antiarrhythmics) and risk factors such as multimorbidity or polypharmacy, that we did find associated to severe falls, may present with lower extremity weakness, disorientation, impaired balance, dizziness, and low body mass.

### Appraisal of potential impact

Attributable fractions afford an idea of the potential absolute impact of these factors on the population. Owing to its extremely high prevalence and strength of association, number of diseases (defined in this analysis as suffering from two or more conditions) accounted for 84% of the fall rate. The attributable fraction for urinary incontinence was likewise very high (49%). It should be noted that these estimates only give an indication of the potential effect of the total elimination of such factors, which is unrealistic but nonetheless helps focus action on modifiable very high-impact factors.

### Strengths and limitations

The generalizability of our results is enhanced by the varied characteristics of the study subjects, the number and variety of facilities included and the very high response rates obtained. Furthermore, our study covered a comprehensive number of relevant variables. Some limitations should be mentioned. First, reverse causation can occur in certain analyses. There can be instances in which the determinant changes due to the fall. For example, depressive symptoms can be a determinant of the occurrence of falls but also can be the consequence of a fall. We believe that this effect is less likely if the fall is without severe consequences. In some cases, however, the effect can theoretically occur even with non-severe falls (e.g. antidepressant, anxiolytics, use of cane, depressive symptoms and insomnia). On the other hand epilepsy can be caused by a fall but it has to be severe (the higher estimate associated to severe fall as compared to non-severe fall is congruent with this possibility). Second, even though the risk period comprised the past 30 days and some variables referred to the preceding seven days or two weeks, we nevertheless believe that they may be plausibly regarded as stable indicators of a previous status. Lastly, some falls are likely to go unreported or unnoticed, leading to an underestimation of the fall rate. Regarding associations with variables the potential misclassification is expected to be non-differential (i.e. the underestimation would affect similarly to the compared groups). It should be mentioned that the potential underreporting bias is likely to be much less important in the case of severe falls.

## Conclusions

We conclude that number of diseases was the most important determinant for multiple falls among our representative population of older persons living in institutions. It is worth mentioning that, although with some exceptions, the effectiveness of interventions for the prevention of falls in care homes is debatable, and number of diseases, a prevalent and difficult-to-mitigate factor in these very aged populations, might go some way to explain this resistance to preventive programs. Other factors – arguably more amenable to control than number of diseases – such as urinary incontinence, antidepressant use, number of medications, and arrhythmias were also clear determinants of accidental falls.

## Competing interests

The authors declare that they have no competing interest.

## Authors’ contributions

JD designed the study and directed its implementation. RPB contributed to the analyses and drafting of the manuscript. EVG had relevant role in the design and implementation of the study and JPC had substantial contribution in the interpretation of data and its critical evaluation. All authors gave final approval for publication. All persons contributing significantly to the work have been listed.

## Pre-publication history

The pre-publication history for this paper can be accessed here:

http://www.biomedcentral.com/1471-2318/13/6/prepub

## Supplementary Material

Additional file 1: Table S1 Distribution of study subjects and proportion of residents with at least 1 fall, by study variables, among institutionalized older adults in Madrid, Spain. Click here for file
