# Supplementary material for: Factors associated with falls among older adults living in institutions
Source: BMC Geriatr. 2013 Jan 15;13:6. doi: 10.1186/1471-2318-13-6 (PMC3566955; doi:10.1186/1471-2318-13-6)
Supplement: Additional file 1: Table S1 — Distribution of study subjects and proportion of residents with at least 1 fall, by study variables, among institutionalized older adults in Madrid, Spain. [file 1471-2318-13-6-S1.pdf]

Table. Distribution of study subjects and proportion of residents with at least 1 fall, by study variables, among institutionalized older adults in Madrid, Spain.

		Fallers		
Grouping	No. (%) [a]	No.	%	95% CI
All	733 (100)	90	12.4	9.4-15.5
Sex				
Women	408 (76)	52	12.5	9.4-16.5
Men	325 (24)	38	12.1	8.1-17.7
Age (years)				
65-74	92 (12)	10	13.9	6.7-26.6
75-84	301 (41)	31	10.3	7.3-14.3
≥ 85	326 (47)	48	14.2	10.7-18.7
Marital status				
Spouse	127 (15)	13	9.6	5.6-16.1
No spouse	576 (85)	75	13.1	10.1-16.9
Educational level				
Less than primary	314 (44)	30	10.4	6.8-15.6
Primary	260 (40)	38	13.8	9.5-19.6
Secondary or higher	96 (16)	16	15.6	9.1-25.3
Facility ownership				
Public	404 (45)	45	12.1	7.8-18.3
Subsidized	78 (9)	14	16.7	12.4-22.0
Private	251 (47)	31	12.0	8.4-16.8
Facility size (no. of beds)				
< 100	154 (28)	15	9.7	5.6-16.2
100–299	283 (39)	35	12.5	8.9-17.4
≥ 300	296 (33)	40	14.7	9.7-21.7
Length of stay (years)				
0-2	255 (36)	28	10.9	7.6-15.3
3-5	214 (31)	26	13.7	8.8-20.6
≥ 6	230 (33)	33	14.2	9.5-20.7
No. of chronic conditions				
0-1	149 (20)	4	2.1	0.6-7.0
2-3	308 (42)	34	10.6	7.0-15.6

≥ 4	276 (37)	52	20.1	15.8-25.2
No. of medications				
0-2	186 (26)	8	3.9	1.7-8.4
3-4	330 (47)	36	10.9	7.6-15.2
≥ 5	189 (27)	44	24.2	18.2-31.5
Antidepressant use				
No	648 (89)	69	10.4	7.9-13.6
Yes	70 (11)	20	29.7	20.8-40.4
Anxiolytic use				
No	567 (76)	64	11.2	8.2-15.2
Yes	151 (24)	25	16.7	10.8-24.8
Hypnotic use				
No	642 (88)	81	13.1	10.0-16.9
Yes	76 (12)	8	8.2	3.7-17.3
Antipsychotic use				
No	633 (87)	76	12.1	9.1-15.7
Yes	85 (13)	13	15.4	8.1-27.3
Functional dependence				
Independent (100 [b])	186 (22)	12	6.1	2.8-13.0
Mild (91-99)	177 (25)	15	8.4	4.4-15.5
Moderate (61-90)	152 (23)	25	15.9	10.9-22.6
Severe (21-60)	91 (15)	25	25.2	17.0-35.6
Total (0-20)	109 (16)	11	10.7	6.3-17.5
Use of cane				
No	516 (74)	58	11.2	8.4-14.8
Yes	204 (26)	30	15.6	10.7-22.3
Use of walker				
No	644 (90)	71	11.2	8.6-14.5
Yes	67 (10)	16	22.9	13.9-35.3
Physical restraint				
No	587 (81)	69	12.0	8.7-16.3
Yes	122 (19)	19	14.2	9.2-21.4
Urinary incontinence				
No	344 (46)	23	6.8	4.3-10.8
Yes	365 (54)	61	16.7	12.8-21.6

Behavioral problems				
No	625 (85)	72	11.6	8.6-15.5
Yes	108 (15)	18	17.1	10.9-25.8
Cognitive status [c]				
Normal (≤2 [d])	269 (54)	39	11.4	6.6-16.2
Mild (3-4)	65 (14)	10	14.4	6.2-22.6
Moderate (5-7)	60 (15)	8	12.5	5.6-19.3
Severe (≥8)	60 (17)	9	16.6	8.7-24.5
Vision impairment				
No/mild	570 (87)	60	11.1	8.3-14.6
Moderate/severe	84 (13)	16	17.9	11.1-27.5
Hearing impairment				
No/mild	549 (86)	61	11.2	8.3-15.0
Moderate/severe	92 (14)	13	14.5	7.4-26.4
Insomnia				
No	491 (69)	55	11.5	8.4-15.5
Yes	222 (31)	33	15.4	10.7-21.6
Self-rated health				
Very good/good	354 (56)	34	10.2	6.8-15.0
Fair/poor/very poor	298 (44)	47	15.8	12.1-20.4
Physician-rated health				
Very good/good	382 (53)	25	7.6	5.0-11.4
Fair/poor/very poor	232 (47)	64	18.2	14.2-23.0
Depressive symptoms (GDS score)				
Normal (0-3)	424 (68)	40	9.8	6.5-14.7
Moderate (4-7)	158 (27)	27	16.5	11.0-24.0
Severe (8-10)	35 (6)	8	26.6	14.5-43.6

Abbreviations: CI = confidence interval; GDS = Geriatric Depression Scale.

[a] Unweighted counts and weighted percentages.

[b] Barthel Index score.

[c] Empirical number of observations and weighted percentages computed through multiple imputation.

[d] Pfeiffer's Short Portable Mental Status Questionnaire score.
